# Differences in Kinematic and Muscle Activity Between ACL Injury Risk and Healthy Players in Female Football: Influence of Change of Direction Amplitude in a Cross-Sectional Case–Control Study

**DOI:** 10.3390/medicina61071259

**Published:** 2025-07-11

**Authors:** Loreto Ferrández-Laliena, Lucía Vicente-Pina, Rocío Sánchez-Rodríguez, Graham J Chapman, Jose Heredia-Jimenez, César Hidalgo-García, José Miguel Tricás-Moreno, María Orosia Lucha-López

**Affiliations:** 1 Unidad de Investigación en Fisioterapia, Spin off Centro Clínico OMT-E Fisioterapia SLP, Universidad de Zaragoza, Domingo Miral s/n, 50009 Zaragoza, Spain; lferrnadez@unizar.es (L.F.-L.); l.vicente@unizar.es (L.V.-P.); r.sanchez@unizar.es (R.S.-R.); jmtricas@unizar.es (J.M.T.-M.); 2 Allied Health Research Unit, School of Health, Social Work and Sport, University of Central Lancashire, Preston PR1 2HE, UK; gchapman2@uclan.ac.uk; 3 Departamento de Educación Física y Deportiva, Universidad de Granada, 51005 Ceuta, Spain; herediaj@ugr.es

**Keywords:** anterior cruciate ligament, biomechanics, electromyography, female football, injury prevention

## Abstract

*Background and Objectives*: Anterior cruciate ligament (ACL) injury rates remain high and have a significant impact on female football players. This study aims to evaluate knee kinematics and lower limb muscle activity in players at risk of ACL injury compared to healthy players through three side-cutting tests. It also investigates how the amplitude of a change in direction influences stabilization parameters. *Materials and Methods*: A cross-sectional case–control study was conducted with 16 second division female futsal players (23.93 ± 5.16 years), divided into injured (*n* = 8) and healthy groups (*n* = 8). Injured players had a history of non-contact knee injury involving valgus collapse, without undergoing surgical intervention. Three change of direction tests, namely the Change of Direction and Acceleration Test (CODAT), Go Back (GOB) test, and Turn (TURN) test, were used for evaluation. The peak and range of knee joint angles and angular velocities across three planes, along with the average rectified and peak envelope EMG signals of the Biceps Femoris (BF), Semitendinosus (ST), Vastus Medialis (VM), and Lateral Gastrocnemius (LG), were recorded during the preparation and load phases. Group differences were analyzed using two-factor mixed-model ANOVA with pairwise comparisons. Statistical significance was set at *p* < 0.05. *Results*: Injured players demonstrated lower external tibial rotation angular velocity and a greater range of motion in tibial external rotation compared to healthy players. Additionally, the injured group showed significantly higher average rectified muscle activity in VM and LG both increased by 4% during the load phase. The CODAT and TURN tests elicited higher BF and VM muscle activity, compared to the GOB test. The TURN test also showed greater extension angular velocity in the sagittal plane. *Conclusions*: The results revealed differences in knee kinematics and muscle activity between players at risk of ACL injury and healthy players, influenced by the amplitude of directional changes. Players altered transverse plane mechanics and increased VM and LG activation during LOAD may reflect a dysfunctional motor pattern, while the greater sagittal plane angular velocity and VM and BF activation from the CODAT and the TURN test highlight their higher potential to replicate ACL injury mechanisms compared to the GOB test.

## 1. Introduction

Anterior cruciate ligament (ACL) injury rates disproportionately affect females, who are 2–6 times more likely to suffer from an ACL injury compared to males with 0.7 injuries per squad per season and 0.1 injuries per 1000 h of play [1,2,3]. ACL injuries are significantly burdensome for the player with, on average, 38 days lost per 1000 h of exposure and 117 days of recovery [1]. Furthermore, 25–35% of players face re-injury within 2–5 years with females having a higher re-injury risk compared to males [4] and with only 81% returning to their prior competition level post-rehabilitation [5,6].

Due to the severity and long-term impact of ACL injuries, current research focuses on identifying ACL risk factors to develop prevention strategies aimed at lowering injury thresholds [7]. Since non-contact mechanisms persist as the primary cause of ACL injuries, kinematic and kinetic analyses have traditionally focused on identifying biomechanical risk factors. Recent research has recognized the importance of addressing valgus collapse, characterized by hip adduction and internal rotation, and knee abduction, incorporating the function analysis of the transverse plane in prevention programs [8,9,10]. While most studies link increased internal tibial rotation to a higher risk of ACL injury, recent research suggests that excessive transverse plane movement may be the primary risk factor [11,12,13,14,15,16,17]. However, to date, there is no consensus as to whether excessive internal or external tibial rotation is a risk factor [16]. Consequently, recent studies advocate for incorporating new outcomes, such as angular velocity, into kinematic analyses, as it reflects the speed of joint movement, closely linked to motor control [11,12,16]. Therefore, it serves to characterize the direction and quality of control, based on movement velocity during the stabilization task.

Sensorimotor control, driven by muscle activity, directly influences the kinematic and kinetic factors associated with ACL injury mechanics [18]. Recent research has demonstrated that altered muscle activity is related to ACL injured risk [10,13,19,20]. Most prevalent injury mechanism occurs during defensive actions, particularly during front-facing pressing situations, where the player must rapidly change direction to follow offensive opponent [21,22]. Therefore, recent studies suggest that functional tasks, such as change of direction tests, can be an effective strategy for assessing risk by replicating the mechanisms of ACL injuries [23,24]. However, it remains unclear which specific amplitude of directional change presents the greatest challenge to knee stabilization, thereby placing the maximum stress on its functional mechanisms [22].

Notably, most ACL stabilization loading occurs in the sagittal plane, primarily involving the hamstrings, quadriceps, and gastrocnemius muscles [13,18,25]. Hamstrings play a crucial role as ACL synergist by counteracting anterior tibial translation during change of direction stabilization manoeuvres [10,13,19]. Previous research highlights that altered hamstring activity, prior to initial ground contact, is associated with an increased risk of ACL injury [10,13,19,20,26]. Semitendinosus (ST) is particularly significant due to its role as a ‘knee adductor’, contributing to medial joint compartment compression and preventing valgus collapse [10,19,26]. Valgus collapse is often associated with increased quadricep activity, which has been suggested to increase anterior shear forces and places excessive strain on the ACL, particularly during the load phase when players re-establish their stability [13,19,26]. Additionally, recent studies have analyzed the role of the gastrocnemius in the stabilization process, identifying its function as ACL antagonist [18,25]. It may perform a posterior displacement of the femur that may contribute to anterior tibial translation, thereby enhancing the action triggered by quadricep contraction during the load phase [18,25].

Given the combined impact of the ACL incidence and its burdensome, along with the persistent sex-related prevalence disparities, improving evaluation strategies in female players remains essential [1,3]. Although previous studies have combined kinematic and motor control assessments, there is still a need to enhance sensitivity of movement quality evaluations and the specificity of muscle activity analysis during tasks that replicate ACL injury mechanisms. In this context, incorporating new kinematic variables, for example, angular velocity, which is more closely linked to motor control, alongside traditional joint angle measurements could enhance assessment specificity [11,12,16]. Similarly, focusing on key muscles such as hamstrings and quadriceps and considering secondary muscles, including gastrocnemius to analyze functionality, may help distinguish motor patterns between players at risk of ACL injury and healthy players [13,19,25]. Therefore, the first objective of this study was to evaluate angular velocity and joint angle kinematics of the knee, as well as muscle activity in hamstrings, quadriceps, and gastrocnemius, both in players at risk of ACL injury and in healthy players during three change of direction tests. The second objective was to evaluate how the amplitude of the change of direction angle involved in each test influences the knee stabilization pattern of the player, based on kinematic and muscle activity variables. It was hypothesized that players at risk of ACL injury will show differences in kinematic and muscle activity patterns compared to healthy players. Additionally, this study suggests that such differences emerge during change of direction tasks and are influenced by the amplitude of directional change.

## 2. Materials and Methods

### 2.1. Participants

Potential participants were identified from professional female futsal teams (Real Federación de Fútbol de Ceuta). They were eligible if they held an active national futsal license, train for over 8 h per week, competed in the Second Spanish National Futsal Division, and actively participated in competitions at the time of the study. Participants were excluded from the study if they had any lower limb injury which may affect the outcomes of this study or had previously received knee surgery and/or any other lower limb surgery.

A cross-sectional case–control study was conducted, and the recruitment was during 2022/2023 season. Participants were allocated to groups based on their clinical history. Injured players were defined as those who had previously sustained a non-contact knee injury resulting from a valgus collapse mechanism, without having undergone ACL surgical intervention. All injuries had to be fully recovered through conservative treatment by the time of the study in accordance with criteria used in previous research [4,9,27,28]. The control group consisted of players that were injury free and had not previously sustained a knee injury. Eight players were allocated in each group. The Research Ethics Committee of the Community of Aragón approved this study (code PI20/127), which adhered to the ethical principles of the Declaration of Helsinki [29]. All participants provided written informed consent prior to data collection commencement. For those participating players who were minors, it was also signed by their legal guardian.

### 2.2. Procedure

**Figure 1:**
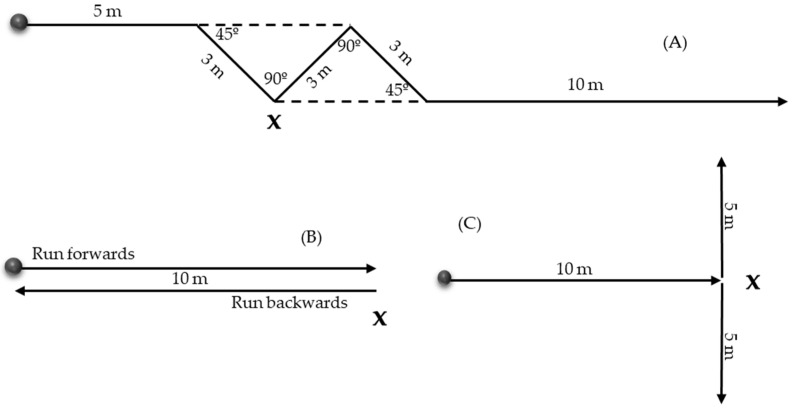
Functional change of direction test: CODAT, GOB test, and TURN test. Starting position is indicated with the dark circle and the cross identifies the change of direction task that was recorded. (**A**) Change of Direction and Acceleration Test (CODAT)—this test combines sprinting mechanics with the stabilization and acceleration required for change of direction movements. It consists of four diagonal change of direction tasks: two at 45° and two at 90°, interspersed with 3 m sprints and culminating in a 10 m sprint. (**B**) Go and Back (GOB) test—this test involves a 10 m frontal sprint at maximum possible speed, followed by deceleration and a final backward sprint. It simultaneously incorporates a change of direction and a deceleration task, both of which are common mechanisms associated with ACL injuries. (**C**) TURN test—this test is a modified version of the T-test. It involves a frontal sprint followed by a single pre-planned 90° change of direction, performed once in each direction.

Participants took part in a single testing session and following a 10 min warm up consisting of mobility exercises and variable intensity running with change of direction drills, were required to complete one trial under three different change of direction tasks: (1) CODAT [27,30,31], (2) GOB [32], and (3) TURN [24,33] (see Figure 1). These tests require performing a change in direction at maximal velocity, but each test involves different amplitudes of directional change. In CODAT and TURN, a 90° change in direction was recorded. However, in CODAT, this occurs after an initial 45° directional change and is followed by two additional consecutive changes in direction, requiring the player to continually adapt their trajectory. In contrast, the TURN test involves a single 90° change of direction, after which the player continues running straight to complete the task. In GOB test, a 180° change in direction was recorded, which includes a forward braking phase followed by running back towards where the participant started the task. Injured participants were instructed to use their injured limb and the healthy participants used their dominant limb as the stance limb for each change of direction test as the support limb in order to replicate the injury mechanism associated with non-contact injuries [21]. Prior to data collection, participants completed a familiarization period to avoid learning bias [27]. Participants were given a unique anonymized study code to minimize any bias during data analysis.

### 2.3. Marker and EMG Sensor Placement

A total of twenty-six markers were placed on the anterior and posterior superior iliac spines, the greater trochanters bilaterally, the medial and lateral femoral epicondyles, and the medial and lateral malleoli. Non-orthogonal tracking clusters comprising four markers were positioned on the lateral thighs and lateral shanks. The feet were modelled as single segments with four markers attached to each foot on the calcanei, first metatarsal, fifth metatarsal, and midfoot. Kinematic data were captured at 250 Hz using a nine-camera 3D motion capture system (Qualisys AB, Göteborg, Sweden).

Surface EMG were recorded using four Trigno Avanti (Delsys Inc., Natick, MA, USA) wireless sensors sampled at 1000 Hz positioned over the Biceps Femoris (BF), ST, Vastus Medialis (VM), and Lateral Gastrocnemius (LG) following the SENIAM guidelines [19,34]. Throughout data collection, regular checks of the signal-to-noise ratio were conducted to ensure good signal quality.

### 2.4. Data Analysis

Marker trajectories and EMG data were exported to C3D and imported into Visual 3D (HAS Motion, Kingston, ON, Canada) for analysis. Kinematic data were filtered using a 4th order zero-lag, 8Hz low-pass Butterworth filter [35]. Knee joint kinematics were calculated from the shank relative to the thigh using an XYZ cardan sequence [36]. Peak knee kinematics in the sagittal, coronal, and transverse planes were extracted [36]. EMG signals were filtered using a second-order Butterworth high-pass filter with a cut-off frequency of 40 Hz to minimize movement artifacts [37] and then full-wave rectified and low-pass filtered with a 15Hz cut-off frequency [37]. The maximum observed signal from the filtered data across all trials and muscles was used to normalized the average and peak EMG signals during the preparation (PREP) phase, defined as 100ms prior to ground contact to the frame immediately before initial contact and the loading (LOAD) phase defined as initial contact to maximum knee flexion [13,38].

### 2.5. Sample Size

The sample size was calculated based on a minimum expected difference of 0.16 (SD 0.11) in BF muscle activity during PREP phase in a change of direction test [38] using the GRANMO 8.0 calculator, considering an alpha risk of 0.05, a beta risk of 0.20, and a two-sided test. A target sample of 16 participants, eight per group, was required.

### 2.6. Statistical Analysis

Shapiro–Wilk tests were performed to explore data normality. For normal distributed data, two-factor mixed method (2 × 2) ANOVA tests were used to explore kinematic differences between groups (injured and healthy) during the three different movement tasks (CODAT, GOB, and TURN). For EMG measures, separate mixed methods ANOVAs were run for the PREP and LOAD phases. For significant main effects, pairwise comparisons were conducted to explore differences between groups and tasks. For significant interactions, separate one-way ANOVAs were performed. The significance level was set at *p* < 0.05 and all statistical analysis were performed using SPSS software v.25 (SPSS Inc., Chicago, IL, USA).

## 3. Results

Eight participants were allocated to the injured group and eight to the healthy group, based on their clinical history. The average age was 23.93 ± 5.16 years with an average height of 1.61 ± 0.05 m; demographic characteristics are summarized in Table 1.

Table 2 shows the descriptive statistics, main effects and interactions for sagittal, coronal, and transverse plane knee kinematics. No differences were reported in the coronal plane for task or group. In the sagittal plane, the mixed methods ANOVA revealed a significant main effect of task for peak knee flexion–extension angular velocity (*p* = 0.049) (Table 2). Post hoc pairwise comparisons showed the CODAT and GOB demonstrated significantly higher knee extension angular velocity compared to TURN task (*p* = 0.035, and *p* = 0.046, respectively) (Table 5).

**Figure 2:**
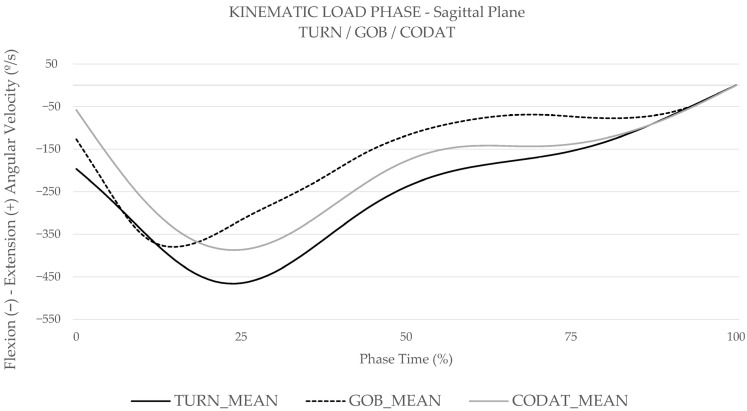
Sagittal plane for angular velocity of TURN, GOB, and CODAT mean values during the LOAD phase.

**Figure 3:**
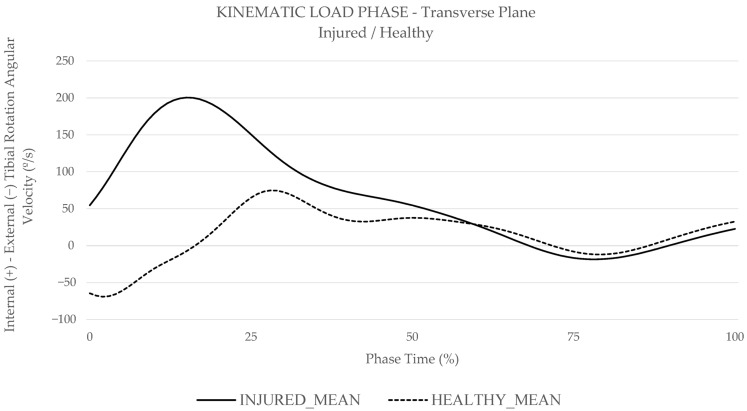
Transverse plane for injured and healthy players based on mean player angular velocity data from functional tests (CODAT, GOB, and TURN) in the CODAT test during the LOAD phase.

In the transverse plane, mixed methods ANOVA showed a significant main effect of group on knee internal–external rotation range of motion (*p* = 0.006) and minimum knee external rotation angular velocity (*p* = 0.034). Post hoc pairwise comparisons revealed that the injured group demonstrated significantly increased knee internal–external rotation range of motion (*p* = 0.006) and significantly decreased external rotation angular velocity (*p* = 0.034). (Table 5). These kinematic post hoc pairwise comparisons were graphically represented in Figure 2, in the sagittal plane for task comparison, and in Figure 3, in the transverse plane for group comparison.

Table 3 and Table 4 show the mixed methods ANOVA results for average and peak muscle activity for the PREP and LOAD phases, respectively. There were no significant differences between groups or the main effect of task for EMG measures during the PREP phase (*p* > 0.05) (Table 3). In the LOAD phase, mixed model ANOVA showed a significant interaction between group and task for peak lateral gastrocnemius muscle activity (*p* = 0.025). Post hoc one-way ANOVA revealed that peak LG muscle activity was significantly higher for the injured compared to the healthy group during the TURN task (*p* = 0.022) (Table 6). There were no between-group differences for either the GOB or CODAT for LG muscle activity (*p* > 0.05) (Table 6).

There were also significant main effects of tasks on average BF muscle activity (*p* = 0.042) between tasks. Post hoc pairwise comparisons that CODAT and TURN tasks significantly increased average BF (*p* = 0.034 and *p* = 0.040, respectively) compared to GOB (Table 4).

There was also significant main effect of group on average VM and peak LG muscle activity (*p* = 0.031 and 0.036, respectively) (Table 3). For group condition, post hoc pairwise comparisons showed that the injured group demonstrated significantly increased average VM and LG muscle activity compared to the healthy group (*p* = 0.031 and *p* = 0.036, respectively) (Table 4).

**Table 5:** Knee kinematics and EMG pairwise comparisons for significant main effects of injured condition and task.

Variable—Knee Kinematic		Mean Difference	*p* Value	95% Confidence Intervals for Differences	
				Lower Bound	Upper Bound
***Sagittal Plane*** *(Significant Main effects of Task)*					
Peak Knee Flexion–Extension Angular Velocity	CODAT and GOB	59.82	0.247	−46.12	165.76
	CODAT and TURN	93.29	0.035 *	3.55	82.00
	GOB and TURN	33.47	0.046 *	2.18	208.61
***Transverse Plane*** *(Significant Main effects of Group)*					
Knee Internal–External Rotation Angle ROM	Injured and Healthy	4.66	0.006 *	1.42	7.89
Minimum Knee External Rotation Angular Velocity	Injured and Healthy	71.54	0.034 *	5.92	137.15
**Variable—Muscle Activity EMG**					
*(Significant Main effects of Task)*					
Average Biceps Femoris Load Phase	CODAT and GOB	0.05	0.034 *	0.00	0.10
	CODAT and TURN	0.01	0.812	−0.05	0.06
	GOB and TURN	−0.05	0.040 *	−0.09	−0.00
Average Vastus Medialis Load Phase	CODAT and GOB	0.04	0.030 *	0.01	0.08
	CODAT and TURN	−0.01	0.711	−0.05	0.04
	GOB and TURN	−0.05	0.046 *	−0.10	−0.00
Peak Vastus Medialis Load Phase	CODAT and GOB	0.24	0.018 *	0.05	0.43
	CODAT and TURN	0.06	0.535	−0.13	0.25
	GOB and TURN	−0.18	0.080	−0.39	0.02
*(Significant Main effects of Group)*					
Average Vastus Medialis Load Phase	Injured and Healthy	0.04	0.031 *	0.00	0.08
Average Lateral Gastrocnemius Load Phase	Injured and Healthy	0.04	0.036 *	0.00	0.08

* denotes significance.

**Table 6:** Pairwise comparison for significant main effect of limb for each task for Peak Lateral Gastrocnemius during the load phase.

Variable *Peak Lateral Gastrocnemius Load Phase*		Mean Difference	*p* Value	95% Confidence Intervals for Differences	
				Lower Bound	Upper Bound
CODAT	Injured and Healthy	−0.17	0.203	−0.45	0.10
GOB	Injured and Healthy	−0.09	0.616	−0.44	0.27
TURN	Injured and Healthy	0.33	0.022 *	0.05	0.60

* denotes significance.

## 4. Discussion

This study investigated the differences in kinematic and muscle activity outcomes associated with ACL injury mechanisms during functional change of direction tasks, comparing players at risk of ACL injury with healthy players. Additionally, it examined whether these differences were influenced by the amplitude of the angle involved in each different change of direction test. The findings support the hypothesis that players at risk of ACL injury, defined as those with a history of valgus collapse-related knee injury, demonstrate altered functional motor pattern compared to healthy players, as reflected by movement quality and motor control. Moreover, both knee kinematics and muscle activity were also influenced by the amplitude of the angle required in each functional change of direction test.

The injured group showed significant differences for kinematics exclusively in the transverse plane, where they exhibited higher internal–external tibial rotation range of motion and decreased external rotation angular velocity compared to the healthy group. This altered kinematic pattern amplifies the rotational load on the ACL, especially in the internal rotation direction, which is critical during pivot shift manoeuvres that involve knee abduction and internal tibial rotation, thereby increasing ACL injury risk [39,40]. This aligns with Bates et al., who explained that the ACL primarily stabilizes anterior tibial translation in the sagittal plane (87%) while also resists torsional forces (13%) from movements in the transverse and coronal planes [39,41]. Additionally, Hewett et al. outlined how axial forces increase compression on the lateral knee side during valgus collapse [42], which combined with the posterior slope of the lateral tibial plateau, enhances internal tibial rotation [15]. This increased internal rotation corresponds with the motor pattern observed in the injured group, highlighting kinematic dysfunctions directly associated with the ACL injury mechanism. Our findings align with these studies, as the injured group demonstrated reduced external tibial rotation angular velocity, particularly during the early LOAD phase (Figure 3), when main stabilization adjustments are essential to avoid excessive strain in the ACL [13]. These are consistent with research that identified greater rotation motion, impaired in internal rotation direction, in players at risk of ACL [12,43,44]. Consequently, the injured group may be more likely to exhibit high risk ACL kinematic profiles, particularly in the transverse plane, compared to the healthy group.

Group differences in muscle activity were observed exclusively during the LOAD phase. Kinematic profile differences, particularly in the transverse plane, may influence muscle activity during load absorption, explaining the lack of differences during the airborne PREP phase. Injured players demonstrated significantly higher average VM and LG muscle activity, 4% more than healthy players, and developed a 33% higher peak LG activity only during the TURN test. Quadricep-dominant strategies during LOAD phase increase anterior tibial shear forces, contributing to excessive ACL strain and higher injury risk [10,13]. As Murphy et al. explains, VM, as a uni-articular muscle, plays a key role in knee stabilization [45]. Therefore, the increased VM activity in the injured group may reflect greater neuromuscular demand for rapid stabilization, potentially resulting from impaired motor control. In addition, LG anatomical position enables posterior femoral translation and posterior knee compression [13,18,25], which, in synergy with quadriceps activity, generates anterior tibial shear forces, further increasing ACL strain [13,18,25]. As noted by Nasseri et al., this interaction reaches its peak early in the LOAD phase, along with the previously mentioned kinematic imbalances [13] (Figure 2 and Figure 3). Our findings highlight this dysfunctional neuromuscular mechanism in the injured group, where elevated VM and LG activation promotes anterior tibial displacement, thereby increasing ACL strain and significantly raising injury risk. This injury pattern, according to Picot et al., could be attributed to muscle compensation related to dysfunctional hamstrings muscle activity, key stabilizers of knee rotation [10,19]. Despite the absence of significant differences in hamstring muscle activity between groups or between BF and ST, the presence of transverse plane kinematic differences suggests that injured players compensate through VM and LG EMG to maintain rotational stability, highlighting a potentially dysfunctional strategy that could underline the increased ACL injury risk. This pattern may raise immediate ACL injury risk and contribute to long-term vulnerability [4,13,19].

Task analysis indicates that the amplitude of directional change significantly impacts kinematic strategies, particularly in the sagittal plane. This highlights this plane load component as the primary involved in change of direction tasks, supporting functional test specificity, as most ACL loading occurs in the sagittal plane during the LOAD phase [13,20,25,39]. The results revealed that both CODAT and GOB exhibited significantly higher knee extension angular velocity compared to TURN. In our study, the maximum knee flexion marks the end of the LOAD phase [13,38,46]. In this way, the high extension angular velocity during CODAT and GOB appears at the end of LOAD phase (Figure 2). According to Thomas et al., increased knee flexion during the LOAD phase aids in achieving an optimal body position at final contact, characterized by a lower center of mass, which enhances force absorption and control of external disturbances [46]. The higher extension angular velocity observed in CODAT and GOB during this phase suggests a more efficient transition from stabilization, i.e., increased then decreased flexion angular velocity, to propulsion, i.e., extension angular velocity. In contrast, TURN appears to present a greater stabilization challenge, potentially delaying this transition and reducing extension angular velocity. Therefore, TURN may better replicate functional injury mechanisms in defense football context, as it allows players to perform the manoeuvre at high intensity, preceded and followed only by straight-line sprinting [22].

Significant differences in muscle activity between tasks were observed only during the LOAD phase. This suggests that the amplitude of directional change during the functional change of direction manoeuvre does not influence muscle activity in the PREP phase. Both CODAT and TURN tasks resulted in higher average BF and VM muscles compared to GOB. Murphy et al. highlight that the hamstrings and quadriceps are the primary controllers of knee stability [45]. This is because most of the knee stabilization load occurs in the sagittal plane, which is the primary axis of action for the hamstrings and quadriceps functioning in an agonist–antagonist balance [10,13]. Therefore, CODAT and TURN, which involve the greater activation of these muscle groups, suggest that a 90° change of direction manoeuvres implicates a greater stabilization challenge for the knee compared to other tasks, such as GOB, that involve other amplitude angles. Supporting this, Markström et al. report that 90° directional changes are associated with more specific and demanding functional patterns than other directional amplitudes [40]. These results, when considered alongside the kinematic data, suggest that tasks involving 90° directional changes may offer a more effective means of evaluating knee stability. Overall, the increased muscle demands and coordination required during these tasks may better reflect functional challenges faced during high-risk movements, such as those related to ACL injury mechanisms. 

Future research should investigate the transferability of these findings to other sports, as change of direction is a common functional task across various disciplines. It would be valuable to examine whether similar kinematic and muscle activity patterns are present in athletes exposed to valgus collapse knee injury mechanisms in different sporting contexts. This exploration could enhance the development of cross-sport prevention strategies. Additionally, further research should focus on the design and implementation of specific training programs based on the kinematic and muscle activity patterns identified in players at risk of ACL injury. These interventions should aim to reduce the magnitude of these risk-related variables, reverse the dysfunctional knee stabilization patterns observed, and ultimately prevent ACL injuries.

### 4.1. Limitations

This study presented some limitations. The assessor(s) in the present study were not blinded during data collection. However, participants were given a unique, anonymized study code to mitigate potential bias during data and statistical analyses. Although this study was powered appropriately, the sample size is small and the results should be interpreted with caution. However, these findings do support the use of kinematics, angular velocity, and muscle activity to explore differences between those at risk of ACL injury and healthy participants during change of direction tests. Additionally, potential covariates such as player age, years of sport participation, and injury type were not included in the a priori statistical plan. Future research may want to appropriately power a study to explore these factors. The methodological limitation of this study is its exclusive focus on knee kinematics, as knee motion is influenced by the kinematics of the entire lower limb. Future research may want to include hip and ankle kinematics and/or muscle activity to gain a more comprehensive understanding of functional differences between players at risk of ACL injury. Furthermore, incorporating gluteal muscle activity analysis would be valuable, as these muscles play a key role in controlling hip movement, which in turn affects knee stability, particularly in the transverse plane.

### 4.2. Clinical Contributions

This study highlights the growing trend of incorporating muscle activity analysis, as neuromuscular factors are related to motor control, into ACL injury risk identification studies. In synergy with kinematics, variables such as angular velocity may provide more sensitive information to identify players at risk of ACL injury, based on the quality of movement analysis. Furthermore, since the study involved active players who had previously suffered an injury due to valgus collapse and had fully recovered, it is plausible that the observed risk patterns reflect dysfunctional compensatory mechanisms. Our findings show that players at risk of ACL injury exhibited greater angular velocity and range of motion in the transverse plane, along with heightened VM and LG muscle activity, compared to healthy players. These alterations in movement quality and motor control reinforce the hypothesis of a dysfunctional compensatory motor pattern. Therefore, these risk patterns should be leveraged to design more targeted injury prevention programs that address these movement strategies and mitigate long-term risk factors.

## 5. Conclusions

This study identified differences in knee angular velocity and muscle activity between players at risk of ACL injury and healthy players. These differences were influenced by the amplitude and direction of the change of direction, indicating that CODAT and TURN tasks, involving 90° directional changes, may offer a more effective means of evaluating knee stability. Players at risk of ACL injury exhibit an increased range of motion and angular velocity in the transverse plane, along with elevated VM and LG muscle activity during the LOAD phase, compared to healthy players. Conversely, the TURN and CODAT tests are characterized by greater angular velocity in the sagittal plane, which is associated with increased activation of the VM and BF muscles. Therefore, the increased angular velocity and range of motion in the transverse plane, along with elevated VM and LG muscle activity during the LOAD phase, may reflect an underlying dysfunctional motor pattern. This highlights the importance of assessing kinematics alongside specific muscle activity during functional tests, replicating ACL injury mechanisms, to better determine player risk profiles and design more effective prevention programs.

## Figures and Tables

**Table 1:** Demographic characteristics of the players.

	Total (*n* = 16)	Injured (*n* = 8)	Healthy (*n* = 8)
Age (years)	23.93 ± 5.16	23.00 ± 4.04	24.75 ± 6.13
Position (Goalkeeper/Back/Wing/Pivot)	3:4:7:2	1:3:3:1	2:1:4:1
Height (cm)	161.24 ± 5.41	162.71 ± 5.87	159.95 ± 4.97
Limb dominance (Right/Left)	13:3	6:2	7:1
Football Experience		15.75 ± 1.98	16.63 ± 1.19
Injury limb (Dominant Limb/Non-Dominant Limb)	4:4	4:4	

**Table 2:** Mean (SDs), and the two-factor mixed linear model statistics for peak sagittal, coronal, and transverse plane knee kinematics during the CODAT, GOB, and TURN tasks.

	CODAT		GOB		TURN		Task *p* Value	Injured *p* Value	Interaction Effect
	Injured	Healthy	Injured	Healthy	Injured	Healthy			
**Variables**									
***Sagittal Plane***									
***Joint Angle***									
Minimum Knee Flexion Angle	19.06 ± 4.49	23.64 ± 8.77	20.10 ± 4.49	21.22 ± 11.96	22.65 ± 2.26	23.83 ± 9.11	0.599	0.307	0.741
Peak Knee Flexion–Extension Angle	−53.64 ± 8.46	54.30 ± 12.04	−62.26 ± 10.18	59.46 ± 8.18	−60.01 ± 10.69	58.41 ± 14.88	0.150	0.696	0.884
Knee Flexion–Extension Angle ROM	−34.59 ± 9.05	30.66 ± 11.17	42.16 ± 10.04	38.23 ± 10.91	37.36 ± 11.57	34.59 ± 9.05	0.134	0.265	0.986
***Angular Velocity***									
Minimum Knee Flexion Angular Velocity	−517.30 ± 127.17	−501.68 ± 126.55	−462.53 ± 64.01	−471.95 ± 11.96	−536.92 ± 109.41	−485.78 ± 124.02	0.380	0.556	0.710
Peak Knee Flexion–Extension Angular Velocity	46.24 ± 140.89	143.81 ± 224.30	11.79 ± 50.65	58.63 ± 77.11	7.32 ± 18.88	−3.85 ± 19.01	0.049 *	0.201	0.164
Knee Flexion–Extension Angular Velocity ROM	562.06 ± 201.87	643.98 ± 197.75	471.74 ± 77.73	524.55 ± 163.86	534.60 ± 110.51	464.36 ± 119.65	0.184	0.628	0.264
***Coronal Plane***									
***Joint Angle***									
Minimum Knee Abduction Angle	−6.67 ± 5.59	−7.87 ± 4.24	−4.42 ± 5.37	−8.80 ± 6.96	−7.07 ± 5.81	−6.65 ± 4.99	0.942	0.289	0.511
Peak Knee Abduction–Adduction Angle	0.90 ± 3.39	−2.25 ± 3.99	2.68 ± 5.72	2.91 ± 7.55	0.67 ± 3.54	1.76 ± 6.80	0.173	0.698	0.385
Knee Abduction–Adduction Angle ROM	7.58 ± 4.37	5.62 ± 2.52	7.09 ± 2.78	11.71 ± 5.50	−7.73 ± 2.92	8.41 ± 3.87	0.152	0.320	0.086
***Angular Velocity***									
Minimum Knee Abduction Angular Velocity	−118.01 ± 76.41	−114.89 ± 37.91	−138.28 ± 77.59	−133.43 ± 45.78	−154.99 ± 137.08	−105.27 ± 51.92	0.672	0.405	0.721
Peak Knee Adduction Angular Velocity	196.10 ± 73.84	140.87 ± 98.07	183.62 ± 73.92	175.43 ± 85.87	244.57 ± 97.19	179.37 ± 91.62	0.388	0.097	0.601
Knee Abduction–Adduction Angular Velocity ROM	314.11 ± 123.82	255.76 ± 127.85	321.90 ± 148.60	308.86 ± 92.26	399.56 ± 223.63	284.64 ± 108.42	0.557	0.145	0.636
***Transverse Plane***									
***Joint Angle***									
Minimum Knee External Rotation Angle	−1.55 ± 8.45	−0.97 ± 8.48	−6.89 ± 9.86	−4.18 ± 9.51	−6.16 ± 9.22	−2.44 ± 7.35	0.386	0.365	0.867
Peak Knee Internal Rotation Angle	11.55 ± 7.96	8.19 ± 7.33	6.54 ± 6.65	7.92 ± 8.63	11.15 ± 6.23	6.19 ± 5.76	0.626	0.269	0.435
Knee Internal–External Rotation Angle ROM	13.11 ± 5.21	9.16 ± 4.40	13.43 ± 5.09	12.10 ± 5.54	17.31 ± 7.37	8.63 ± 5.14	0.558	0.006 *	0.223
***Angular Velocity***									
Minimum Knee External Rotation Angular Velocity	−96.61 ± 92.06	−164.32 ± 145.82	−173.90 ± 120.09	−254.06 ± 134.48	−109.27 ± 58.56	−176.00 ± 96.89	0.129	0.034 *	0.983
Peak Knee Internal Rotation Angular Velocity	253.16 ± 155.06	215.68 ± 126.86	270.14 ± 124.14	233.24 ± 115.39	366.58 ± 200.39	215.80 ± 88.08	0.552	0.071	0.461
Knee Internal–External Rotation Angular Velocity ROM	349.77 ± 225.48	380.00 ± 249.73	444.04 ± 116.70	487.30 ± 221.62	475.85 ± 206.39	391.81 ± 141.91	0.411	0.952	0.564

* denotes significance.

**Table 3:** Mean (SDs) and the two-factor mixed linear model statistics for peak and average muscle activity for Biceps Femoris, Semitendinosus, Vastus Medialis, and Lateral Gastrocnemius during the CODAT, GOB and TURN during preparation phase.

	CODAT		GOB		TURN		Task *p* Value	Group *p* Value	Interaction Effect
	Injured	Healthy	Injured	Healthy	Injured	Healthy			
**Variables**									
Average Biceps Femoris	0.14 ± 0.05	0.13 ± 0.11	0.12 ± 0.04	0.10 ± 0.07	0.15 ± 0.05	0.14 ± 0.12	0.326	0.483	0.893
Peak Biceps Femoris	0.63 ± 0.24	0.59 ± 0.37	0.51 ± 0.12	0.38 ± 0.25	0.68 ± 0.26	0.53 ± 0.42	0.122	0.206	0.842
Average Semitendinosus	0.17 ± 0.09	0.15 ± 0.05	0.19 ± 0.05	0.16 ± 0.06	0.19 ± 0.08	0.14 ± 0.07	0.791	0.097	0.887
Peak Semitendinosus	0.71 ± 0.33	0.75 ± 0.23	0.68 ± 0.20	0.63 ± 0.22	0.78 ± 0.24	0.63 ± 0.18	0.656	0.403	0.576
Average Vastus Medialis	0.11 ± 0.06	0.08 ± 0.05	0.08 ± 0.03	0.07 ± 0.04	0.10 ± 0.05	0.07 ± 0.05	0.335	0.092	0.867
Peak Vastus Medialis	0.49 ± 0.18	0.43 ± 0.31	0.47 ± 0.21	0.33 ± 0.18	0.53 ± 0.23	0.46 ± 0.25	0.420	0.197	0.850
Average Lateral Gastrocnemius	0.08 ± 0.06	0.08 ± 0.06	0.06 ± 0.04	0.07 ± 0.04	0.05 ± 0.02	0.05 ± 0.04	0.217	0.618	0.959
Peak Lateral Gastrocnemius	0.40 ± 0.18	0.48 ± 0.31	0.26 ± 0.19	0.35 ± 0.25	0.38 ± 0.25	0.31 ± 0.29	0.274	0.612	0.633

**Table 4:** Mean (SDs) and the two-factor mixed linear model statistics for peak and average muscle activity for Biceps Femoris, Semitendinosus, Vastus Medialis, and Lateral Gastrocnemius during the CODAT, GOB, and TURN during load phase.

	CODAT		GOB		TURN		Task *p* Value	Group *p* Value	Interaction Effect
	Injured	Healthy	Injured	Healthy	Injured	Healthy			
**Variables**									
Average Biceps Femoris	0.16 ± 0.06	0.13 ± 0.09	0.10 ± 0.03	0.08 ± 0.07	0.15 ± 0.04	0.13 ± 0.08	0.042 *	0.208	0.884
Peak Biceps Femoris	0.78 ± 0.25	0.69 ± 0.31	0.59 ± 0.25	0.47 ± 0.40	0.71 ± 0.26	0.67 ± 0.34	0.188	0.376	0.946
Average Semitendinosus	0.11 ± 0.06	0.14 ± 0.07	0.10 ± 0.02	0.10 ± 0.05	0.13 ± 0.07	0.11 ± 0.05	0.264	0.755	0.653
Peak Semitendinosus	0.64 ± 0.35	0.74 ± 0.24	0.52 ± 0.12	0.63 ± 0.28	0.59 ± 0.27	0.73 ± 0.26	0.395	0.143	0.969
Average Vastus Medialis	0.20 ± 0.05	0.12 ± 0.07	0.13 ± 0.05	0.10 ± 0.07	0.17 ± 0.06	0.16 ± 0.09	0.052 *	0.031 *	0.153
Peak Vastus Medialis	0.91 ± 0.14	0.73 ± 0.31	0.68 ± 0.25	0.49 ± 0.32	0.71 ± 0.23	0.81 ± 0.32	0.051 *	0.253	0.245
Average Lateral Gastrocnemius	0.15 ± 0.07	0.14 ± 0.07	0.12 ± 0.07	0.08 ± 0.04	0.15 ± 0.08	0.08 ± 0.04	0.174	0.036 *	0.433
Peak Lateral Gastrocnemius	0.69 ± 0.28	0.86 ± 0.23	0.52 ± 0.31	0.60 ± 0.35	0.79 ± 0.26	0.46 ± 0.25	0.096	0.770	0.025 *

* denotes significance.

## Data Availability

The datasets presented in this study are available on request from the corresponding author. All data covered by this study are included in this manuscript.

## Abbreviations

The following abbreviations are used in this manuscript:

ACL	Anterior Cruciate Ligament
EMG	Electromyography
ST	Semitendinosus
BF	Biceps Femoris
VM	Vastus Medialis
LG	Lateral Gastrocnemius
PREP	Preparation phase
LOAD	Load phase
